# Magnetic Circular Dichroism of Luminescent Triarylmethyl Radicals

**DOI:** 10.1021/acs.jpclett.4c02793

**Published:** 2024-11-14

**Authors:** Yohei Hattori, Daiya Suzuki, Wataru Ota, Tohru Sato, Gwénaël Rapenne, Yoshitane Imai

**Affiliations:** † Division of Materials Science, Nara Institute of Science and Technology, 8916-5 Takayama, Ikoma, Nara 630-0192, Japan; ‡ Department of Applied Chemistry, Faculty of Science and Engineering, Kindai University, 3-4-1 Kowakae, Higashi-Osaka, Osaka 577-8502, Japan; § Fukui Institute for Fundamental Chemistry, Kyoto University, Takano-Nishibiraki-cho, 34-4, Kyoto 606-8103, Japan; ∥ Department of Molecular Engineering, Graduate School of Engineering, Kyoto University, Nishikyo-ku, Kyoto 615-8510, Japan; ⊥ CEMES-CNRS, Université de Toulouse, CNRS, 29 Rue Marvig, F-31055 Toulouse Cedex 4, France

## Abstract

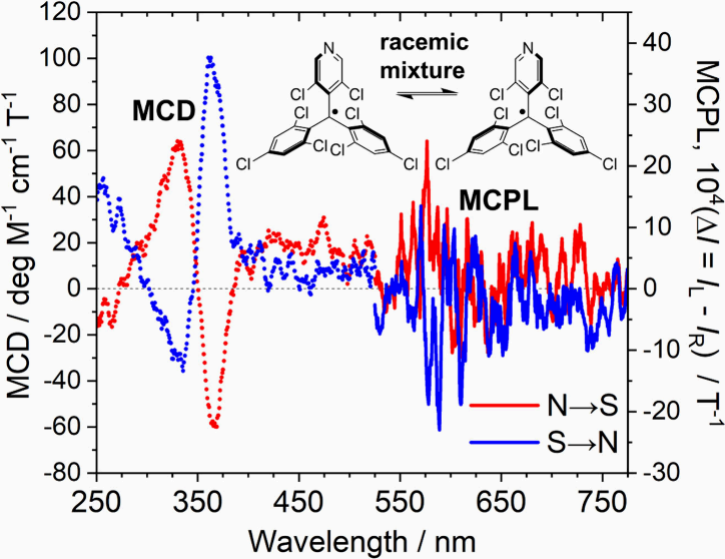

Stable triarylmethyl radicals are the most common carbon radical building blocks and have recently attracted much attention for their luminescent properties. However, magnetic circular dichroism (MCD) discovered by Michael Faraday and magnetic circularly polarized luminescence (MCPL) have not been observed for simple triarylmethyl radicals, probably due to their photodegradability. Here we report the first observation of MCD and MCPL of triarylmethyl radicals in solution using racemic mixtures of (3,5-dichloro-4-pyridyl)bis(2,4,6-trichlorophenyl)methyl radical (PyBTM) and (3,5-difluoro-4-pyridyl)bis(2,4,6-trichlorophenyl)methyl radical (F_2_PyBTM), which are much more photostable than simple triphenylmethyl radical derivatives. Faraday *B* terms, which are at the origin of magnetic dichroism in nondegenerate systems, were calculated using TD-DFT, and the line shape of MCD spectra was well reproduced. This study provides new circular dichroism properties for luminescent triarylmethyl radicals in solution without separating enantiomers and also clarifies the origin of magnetic circular dichroism properties of stable organic radicals for the first time.

Triarylmethyl radicals protected by halogen atoms are the most common form of stable carbon radical.^1^ Three aryl rings are conjugated to the central α-carbon but tilted with respect to the sp^2^ plane with the radical center for steric reasons. Thus, the shapes of these radicals are typically propeller-like.

One of the features commonly found in these radicals is luminescence.^2^ The emission of these radicals is unique compared to that of common closed-shell molecules in that it is a fluorescence from the doublet excited state to the doublet ground state. Spin-multiplicity is particularly important for applications in electroluminescence (EL), and an efficient organic light-emitting diode (OLED) has been obtained.^3^ Unlike the lowest triplet excited state in the closed-shell molecules, there are no spin-forbidden excited states lower than the lowest spin-allowed excited state. Unique photophysical properties such as the specific heavy-atom effect^4,5^ and magnetic-field effect on luminescence (magnetoluminescence)^6,7^ are also observed.

In 2019, Veciana and co-workers separated the left- and right-handed enantiomers of propeller-like triarylmethyl radicals (Figure 1), tris(2,4,6-trichlorophenyl)methyl radical (TTM)^8^ and perchlorotriphenylmethyl radical (PTM)^9,10^ by using chiral stationary phase (CSP) HPLC.^11^ They first reported electronic circular dichroism (ECD) and circularly polarized luminescence (CPL) of both (*P*)- and (*M*)-enantiomers of these radicals at −20 °C, since they racemize at room temperature with a racemization barrier of 20 kcal mol^–1^. They also prepared the tris(2,4,6-tribromophenyl)methyl radical (TTBrM) without any racemization up to 60 °C.^12^

**Figure 1:**
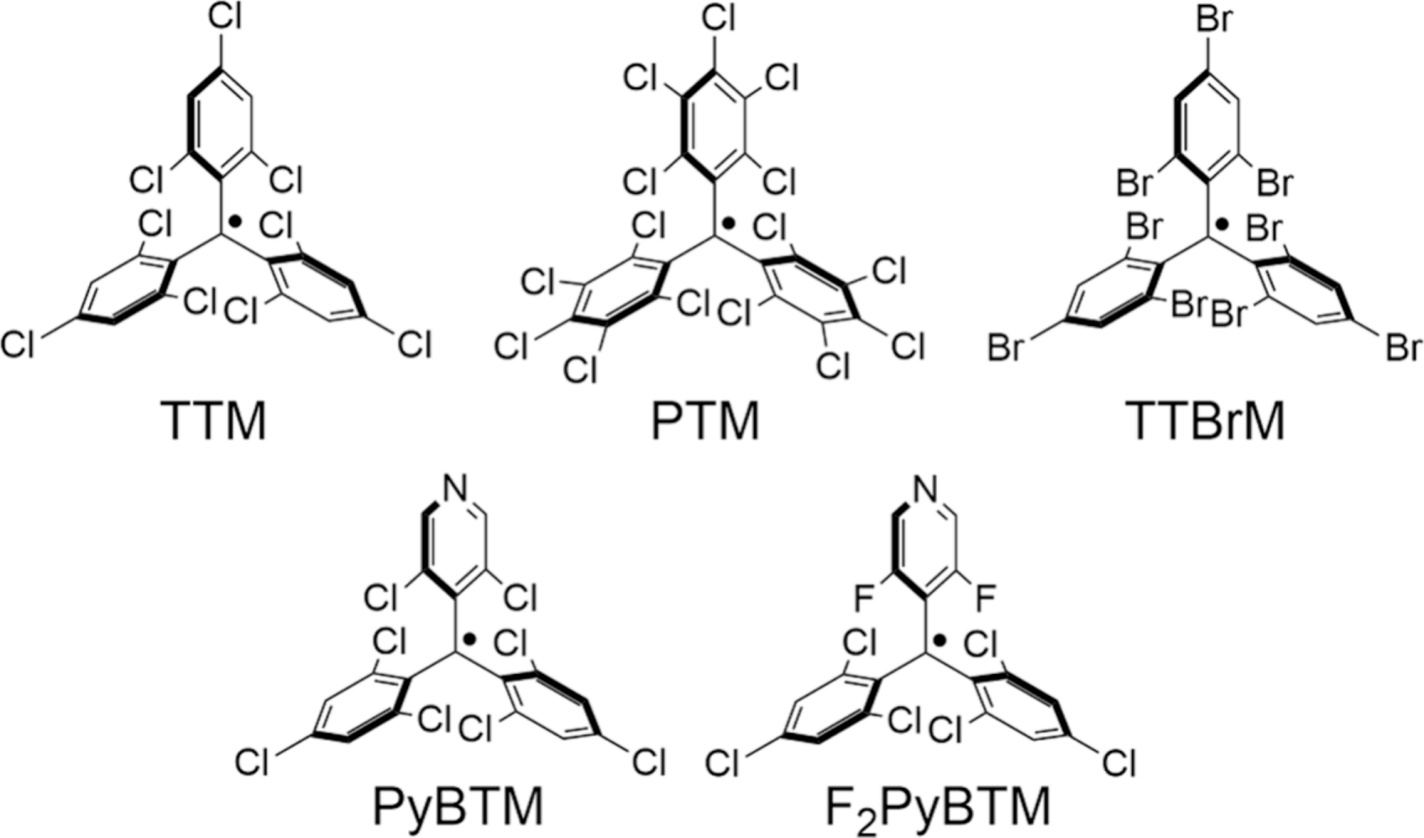
Structures of the (*P*)-enantiomers of TTM, PTM, TTBrM, PyBTM and F_2_PyBTM.

To observe circular dichroism, CSP-HPLC is an expensive option, and subsequent racemization is still a major problem for most triarylmethyl radicals. Circular dichroism of symmetric materials induced by a longitudinal external magnetic field was discovered by Michael Faraday,^13^ and MCD of organic π-systems such as porphyrins, phthalocyanines,^14^ and heme proteins^15^ has been used to study the electronic structures. Circular dichroism in luminescence under longitudinal external magnetic field, called MCPL, is also observed for many basic luminescent organic molecules.^16^ In this paper, we describe the MCD and MCPL of simple triarylmethyl radicals in organic solution at room temperature and discuss their origins.

In this study, we used triarylmethyl radicals, PyBTM^17^ and F_2_PyBTM,^18^ for the measurements of MCD and MCPL. Their absorption and emission properties resemble those of TTM and PTM, which are persistent radicals in the dark but decompose quickly under photoirradiation.^19^ PyBTM and F_2_PyBTM are much more (>70 times) photostable than TTM in dichloromethane and therefore suitable for optical measurements. PyBTM has also been used for some photophysical measurements such as the coherent coupling between spin ensembles^20^ and photoluminescence anisotropy amplified by exciton funneling.^21^

MCD spectra of the triarylmethyl radicals in dichloromethane were measured by using a JASCO J-820 circular dichroism spectrometer with a 0.8 T permanent magnet (Figure 2). As a result of 32 times accumulation of measurements with concentrated solution (∼1 × 10^–3^ M), the visible absorption bands of PyBTM and F_2_PyBTM (550–425 nm) showed weak positive MCD. PyBTM indicated a negative MCD peak at 368 nm and a positive MCD peak at 334 nm. F_2_PyBTM showed a smaller negative MCD signal at 355 nm and a positive signal at 328 nm. The positions of the negative peaks were near the absorption maxima of PyBTM and F_2_PyBTM at 370 and 351 nm, respectively.

**Figure 2:**
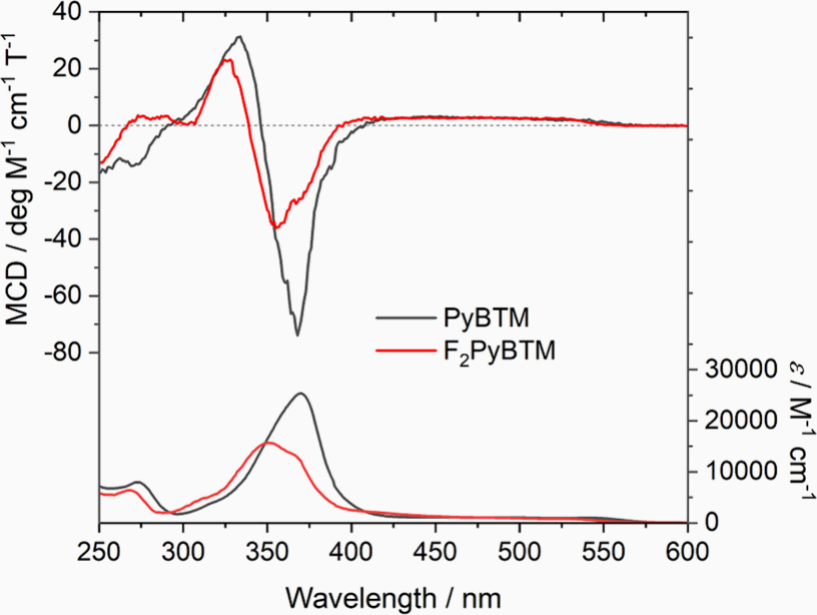
MCD spectra of PyBTM (in black, 1.3 × 10^–3^ M (250–330, 390–420 nm), 5.6 × 10^–4^ M (330–390 nm), 8.0 × 10^–3^ M (420–600 nm)) and F_2_PyBTM (in red, 1.1 × 10^–3^ M (250–330, 380–420 nm), 5.9 × 10^–4^ M (330–380 nm), 9.7 × 10^–3^ M (420–600 nm)) in dichloromethane under 0.8 T (N-up). Absorption coefficients are shown at the bottom.

The intensity of MCD depends on Faraday *A*, *B*, and *C* terms, although only the *B* term is involved in a nondegenerate electronic system.^13^ The *B* term from ground electronic state *m* to excited electronic state *n* is given by^22−24^
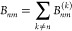
1
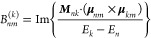
2where *E*_*k*_ – *E*_*n*_ is the energy difference between excited states *k* and *n*, ***M***_*nk*_ is the magnetic dipole moment between excited states, and **μ**_*nm*_ and **μ**_*km*_ are the electric dipole moments between excited and ground states. The MCD spectrum can be calculated from^22−24^
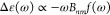
3where ω is the photon frequency and *f*(ω) is the line shape function, which was expressed by the Gaussian function in this study.

The ground and excited electronic structures of PyBTM and F_2_PyBTM, which had *C*_*2*_ symmetry at the ground state, were calculated at the M06-2X/6-31G(d,p) and TD-M06-2X/6-31G(d,p)^25^ functionals within the Tamm-Dancoff approximation, respectively. The solvent effect of dichloromethane was considered using a polarizable continuum model (PCM).^26,27^ We note that the M06-2X functional well reproduced the absorption spectral line shape of PyBTM in both the visible and near-UV regions, compared to the B3LYP functional (Figure S1). The electronic structure was computed using Gaussian 16 Revision C.01.^28^ Magnetic and electric dipole moments were computed using MultiWfn 3.8.^29^ The active space of the excited states was taken from D_1_ to D_50_.

The DFT calculations attributed the weak visible absorption band of PyBTM and F_2_PyBTM to D_0_ → D_1_, which mainly consisted of the β-HOMO → β-LUMO transition for PyBTM and the β-HOMO–1 → β-LUMO transition for F_2_PyBTM (Figure 3, Tables S1–S2).^17,18^ This transition had a small oscillator strength due to the nearly 3-fold molecular symmetry.^30^ The strong near-UV absorption bands were attributed to D_0_ → D_8_ and D_0_ → D_9_, which mainly consisted of the transitions from the α-HOMO to the pseudodegenerate α-LUMO and α-LUMO+1 (Figure 3a, Tables S1–S2).

**Figure 3:**
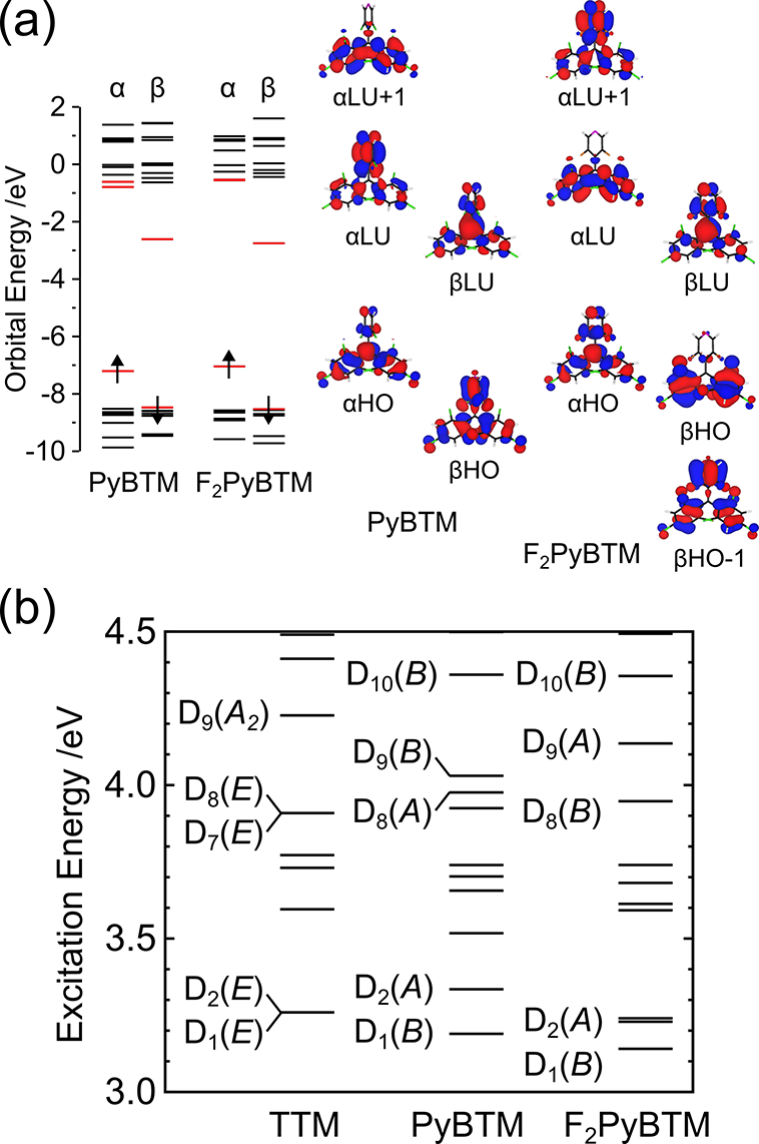
(a) Calculated orbital levels and molecular orbitals (isosurface value: 3 × 10^–2^ a.u.) of PyBTM and F_2_PyBTM at the D_0_ optimized structure. (b) Calculated energy levels of excited states for TTM, PyBTM, and F_2_PyBTM at the D_0_ optimized structure.

The calculated MCD spectra of PyBTM and F_2_PyBTM well reproduced the line shape of the experimental spectrum (Figure 4). The *B* terms of D_8_ and D_9_, which contributed to the negative and positive MCD, respectively, were large. This was attributed to the large electric transition dipole moments of D_0_ → D_8_ and D_0_ → D_9_ and strong magnetically induced mixing between D_8_ and D_9_ (Tables S3–S4). The strong mixing arose from the pseudodegeneracy of D_8_ and D_9_, i.e., energetically close excited states, which originated from the symmetry breaking of degenerate D_7_ and D_8_ of TTM with *D*_*3*_ symmetry (Figure 3b, organic molecules with degeneracy show strong MCD^13−15^). The *B* term of D_10_, which corresponded to D_9_ of TTM, also contributed to the positive MCD because of the mixing with D_8_ and D_9_ (Tables S3–S4). Thus, it was found that the strong MCD was induced by the optical absorption into the pseudodegenerate excited states with large electric dipole moments to the ground state.

**Figure 4:**
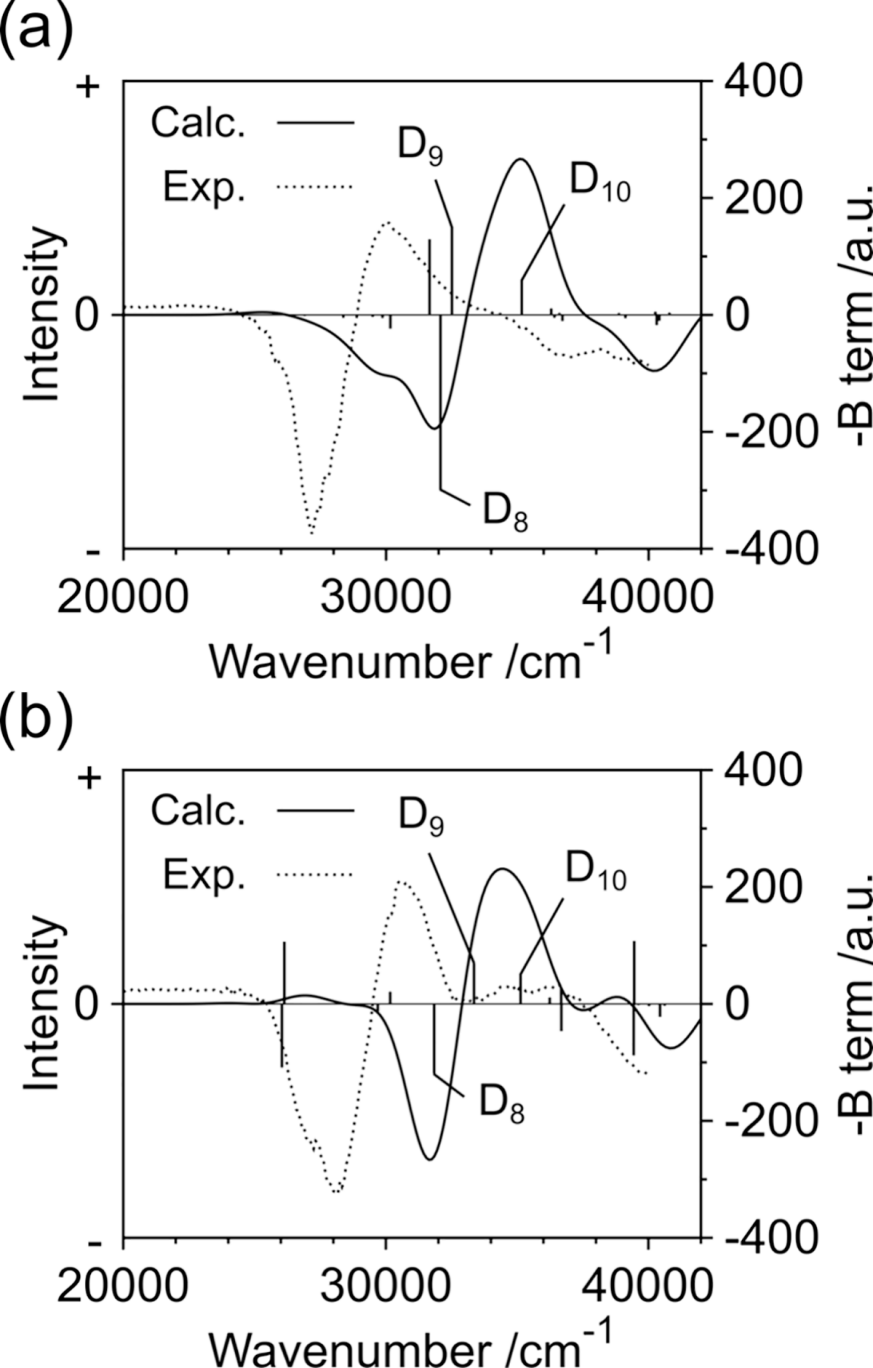
Calculated N-up MCD spectra of (a) PyBTM and (b) F_2_PyBTM with a line width of the Gaussian function 1000 cm^–1^. Vertical lines represent the Faraday *B* terms.

On the other hand, the *B* terms in the visible region were small except for a few excited states (Figure 4 and Figure S2). The slight negative value of the *B* term of D_1_ is consistent with the experimental results, but the accuracy of calculations for small values cannot be guaranteed. The *B* term of D_1_ mainly originated from the mixing with the energetically high-excited states having large electric dipole moments (Tables S3–S4).

MCPL spectra of the triarylmethyl radicals in dichloromethane were measured using a JASCO CPL-300 spectrometer with a 1.7 T permanent magnet (Figure 5). MCD spectra measured using a JASCO J-1700 Circular Dichroism spectrometer with the same magnet are shown in Figure S3. Although the MCD signal of the D_1_ excited state absorption was very weak, positive MCPL signals were shown when Faraday-type N → S (N-up) geometry was employed, and negative MCPL signals were shown when Faraday-type S → N (S-up) geometry was employed. The noises in the spectra could not be eliminated due to the small fluorescence quantum yields of PyBTM (Φ_f_ = 2%) and F_2_PyBTM (Φ_f_ = 4%) and the limited number of accumulations due to the photodegradation by the strong excitation light. Also, the spectral line shapes of MCPL in the visible region might be attributed to the vibronic progressions,^31^ and these effects need to be considered for detailed discussion. Although the line shape is unclear, it is important to note that the sign of the signal was reproducible after repeated similar experiments (Figure S4). The magnet of the CD spectrometer (Figure 1) was fixed to N-up and the sign of MCD corresponding to the D_1_ excited state (weak visible band) agreed with the sign of MCPL. The Faraday *B* terms of D_0_ → D_1_ and D_1_ → D_0_ are small but certainly present. Importantly, CPL was observed without separating the enantiomers.

**Figure 5:**
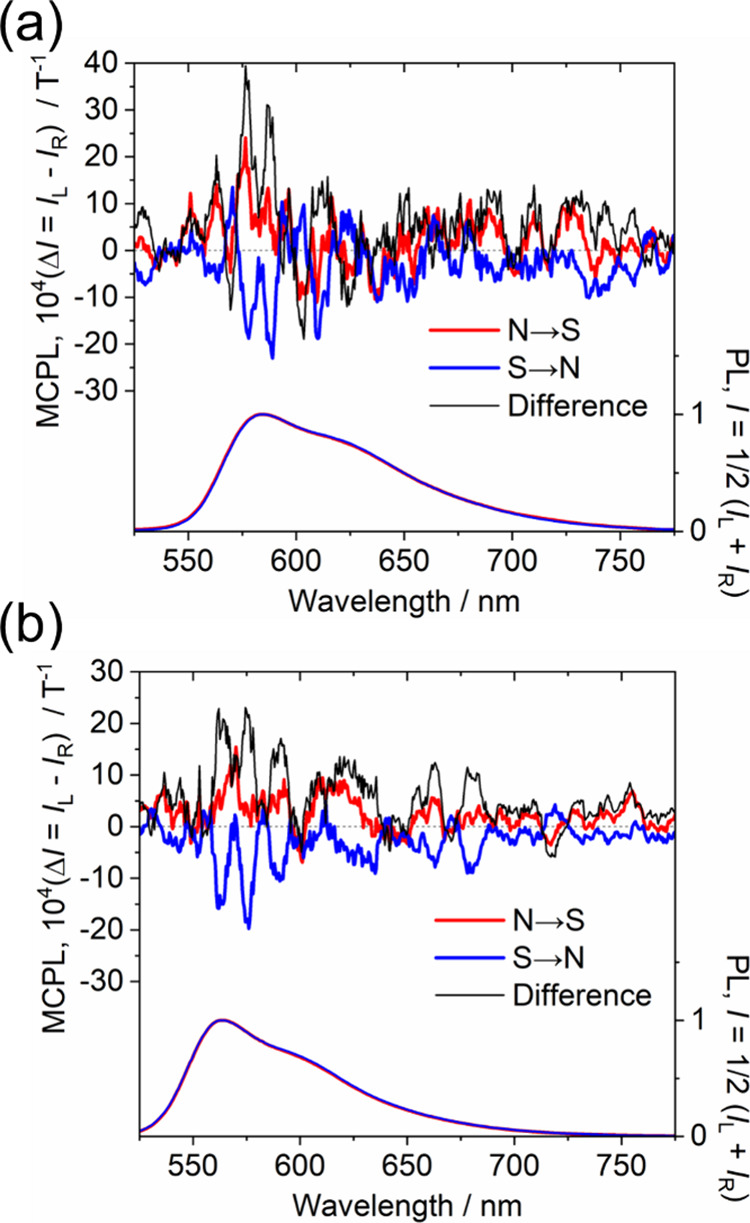
MCPL spectra of (a) PyBTM and (b) F_2_PyBTM in dichloromethane (1.0 × 10^–3^ M) under N-up (red lines) and S-up (blue lines) Faraday geometry under 1.7 T. *I*_L_ and *I*_R_ are the intensities of the left- and right-handed MCPL, respectively. Photoluminescence spectra under a magnetic field are shown at the bottom.

Electron-donor substituents are known to drastically increase the photoluminescence quantum yield of triarylmethyl radicals. For such brightly luminescent donor-radical acceptor systems, CPL properties were induced by a magnetic field, supramolecular assembly and chiral liquid crystal encapsulation.^32^ A chiral donor-radical acceptor system using pillar[5]arene was reported to exhibit CPL.^33^

We also tried to measure MCPL spectra of some donor-PyBTM systems such as Mes_2_F_2_PyBTM^34^ and the donor-bridged diradical system PyBTM-(Hex_2_Ph)PyBTM;^35^ however, MCPL in solution was not detected similarly to donor-TTM or donor-PTM systems in hexane solution.^32^ It is plausible that the rotation of the single bond between the donor and the radical cancels out the CPL properties in solution.

In conclusion, MCD and MCPL of simple triarylmethyl radicals were observed for the first time in solution, and the signals were explained by Faraday *B* terms calculated using DFT. The MCD signal arising from the α-HOMO to the pseudodegenerate α-LUMO and α-LUMO+1 transitions has the potential to give information on the electronic symmetry of these radicals. The ability to induce CPL using only a magnetic field, without requiring enantioenriched samples, opens up new possibilities for luminescent radicals, which are unique as magnetic organic molecules.

## Supporting Information Available

Calculated absorption spectra and Faraday *B* terms, MCD and MCPL spectra under 1.7 T, TD-DFT data tables (PDF)
