# Supplementary material for: Magnetic Circular Dichroism of Luminescent Triarylmethyl Radicals
Source: J Phys Chem Lett. 2024 Nov 14;15(47):11696–700. doi: 10.1021/acs.jpclett.4c02793 (PMC11613632; doi:10.1021/acs.jpclett.4c02793)
Supplement: Supplementary file 1 — jz4c02793_si_001.pdf [file jz4c02793_si_001.pdf]

Supporting Information

Magnetic Circular Dichroism of Luminescent Triarylmethyl Radicals

Yohei Hattori,*[a] Daiya Suzuki, [b] Wataru Ota,*[c,d] Tohru Sato, [c,d] Gwénaël Rapenne [a,e] and Yoshitane Imai *[b]

a. Division of Materials Science, Nara Institute of Science and Technology, 8916-5 Takayama, Ikoma, Nara, 630-0192, Japan.

b. Department of Applied Chemistry, Faculty of Science and Engineering, Kindai University, 3-4-1 Kowakae, Higashi-Osaka, Osaka, 577-8502, Japan.

c. Fukui Institute for Fundamental Chemistry, Kyoto University, Takano-Nishibiraki-cho, 34-4, Kyoto, 606-8103, Japan.

d. Department of Molecular Engineering, Graduate School of Engineering, Kyoto University, Nishikyo-ku, Kyoto 615-8510, Japan.

e. CEMES-CNRS, Université de Toulouse, CNRS, 29 Rue Marvig, F-31055 Toulouse Cedex 4, France

Content

Figure S1 Calculated absorption spectrum of PyBTM using M06-2X and B3LYP functionals.

Figure S2 Calculated Faraday B terms of PyBTM and F_2PyBTM in the visible region.

Figure S3 MCD spectra of PyBTM and F_2PyBTM in dichloromethane under 1.7 T.

Figure S4 Repeated MCPL spectra of PyBTM and F_2PyBTM in dichloromethane under 1.7 T.

Table S1 Excited states of PyBTM at the D_0 optimized structure.

Table S2 Excited states of F_2PyBTM at the D_0 optimized structure.

Table S3 Calculated Faraday B terms of electronic transition from m (D_0) to n for PyBTM.

Table S4 Calculated Faraday B terms of electronic transition from m (D_0) to n for F_2PyBTM.

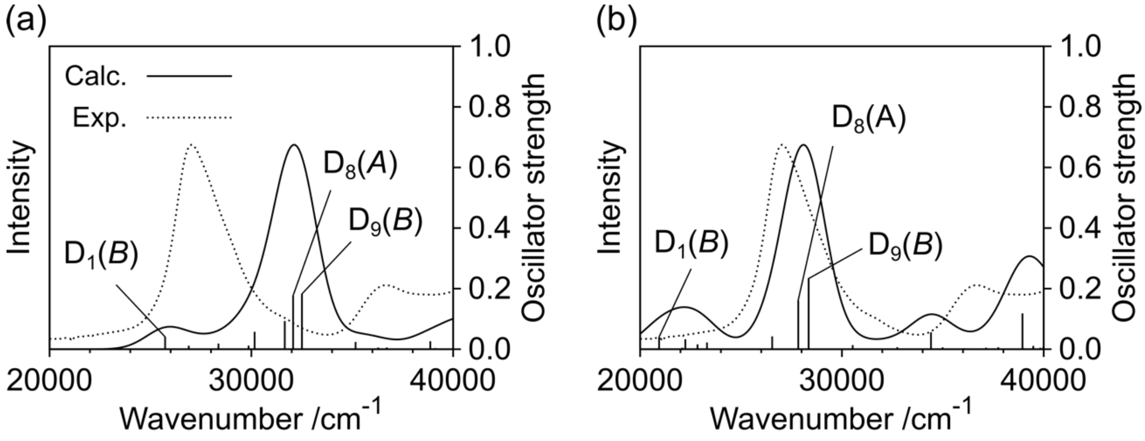

Figure S1. Calculated absorption spectrum of PyBTM using the (a) M06-2X and (b) B3LYP functionals. The vertical lines represent the oscillator strengths. The absorption spectrum was computed by broadening the oscillator strengths using the Gaussian function with a linewidth of 1000 cm^{-1}.

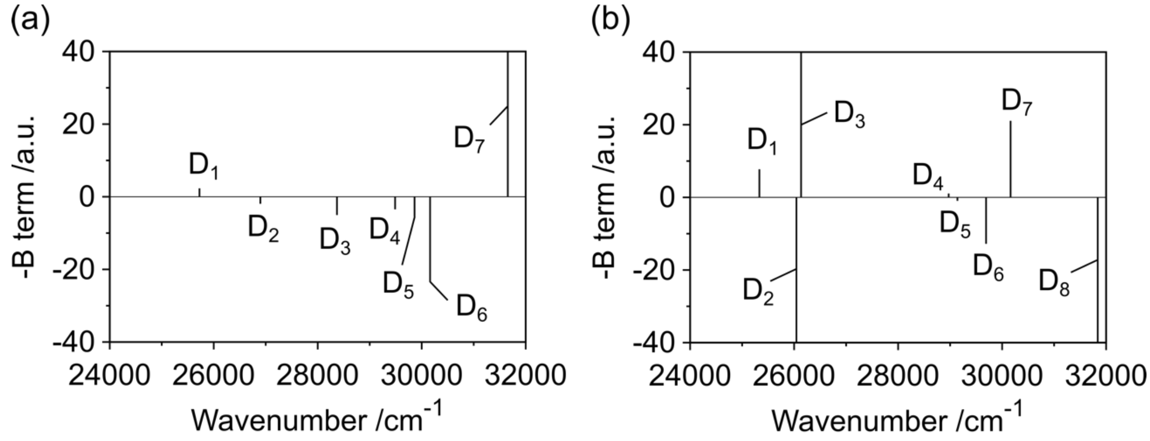

Figure S2. Calculated Faraday B terms of (a) PyBTM and (b) F_2PyBTM in the visible region.

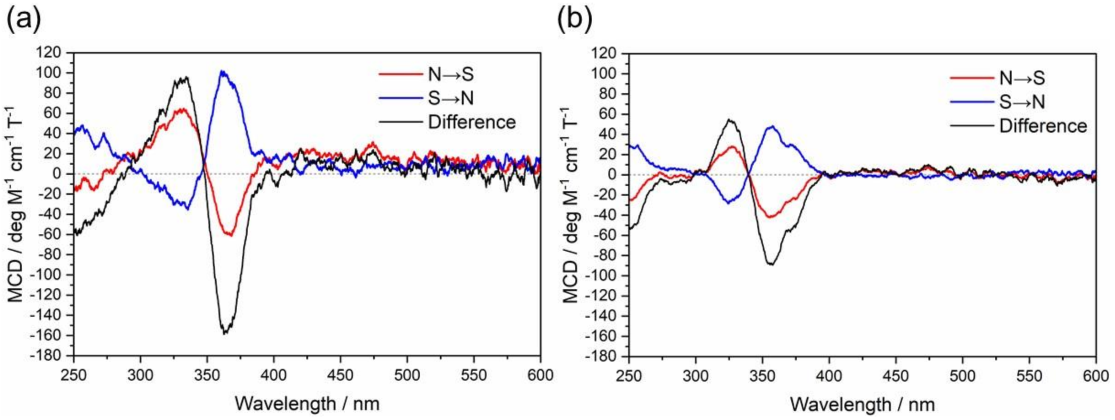

Figure S3. MCD spectra of (a) PyBTM (2×10^{-5} M) and (b) F_2PyBTM (5×10^{-5} M) in dichloromethane under N-up (red lines) and S-up (blue lines) Faraday geometry under 1.7 T.

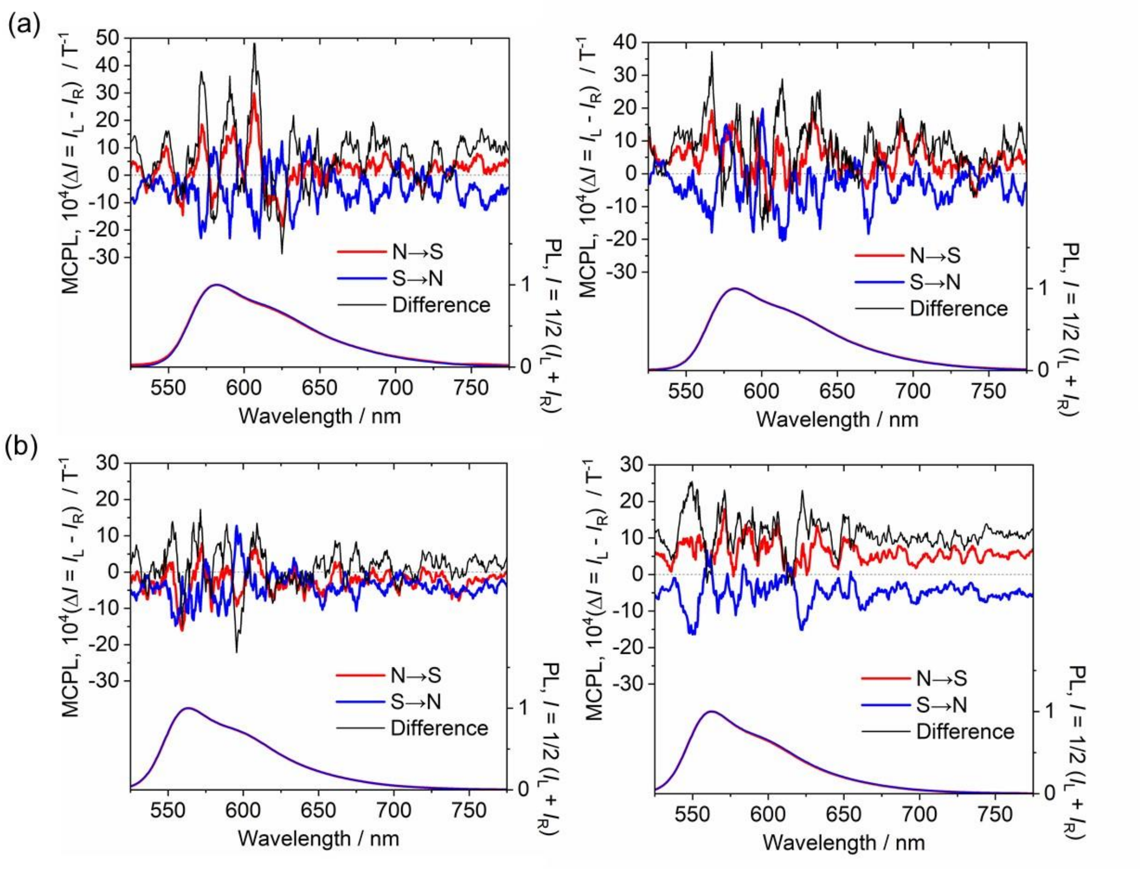

Figure S4. Repeated MCPL spectra of (a) PyBTM (1×10^{-3} M) and (b) F_2PyBTM (1×10^{-3} M) in dichloromethane under N-up (red lines) and S-up (blue lines) Faraday geometry under 1.7 T.

Table S1. Excited states of PyBTM at the D_0 optimized structure. f denotes the oscillator strength.

State	Excitation Energy /eV	/nm	f	Major Configurations (Expansion Coefficients)
D_1 (*B*)	3.189	388.71	0.0407	βHO-2→ βLU (0.37865)
				βHO→ βLU (0.82587)
D_2 (*A*)	3.334	371.79	0.0119	βHO-4→ βLU (0.69887)
				βHO-1→ βLU (-0.52975)
D_3 (*B*)	3.517	352.49	0.0187	βHO-2→ βLU (0.81312)
				βHO→ βLU (-0.41588)
D_4 (*A*)	3.656	339.11	0.0007	βHO-4→ βLU (0.48845)
				βHO-1→ βLU (0.77346)
D_5 (*B*)	3.702	334.88	0.0121	βHO-3→ βLU (0.90101)
D_6 (*A*)	3.739	331.56	0.0576	αHO→ αLU (0.62684)
				βHO-5→ βLU (0.56729)
D_7 (*B*)	3.924	315.90	0.0932	αHO→ αLU+1 (0.61152)
				βHO-6→ βLU (-0.52746)
				βHO-6→ βLU+1 (0.33657)
D_8 (*A*)	3.976	311.82	0.1762	αHO→ αLU (-0.60405)
				βHO-5→ βLU (0.62955)
D_9 (*B*)	4.030	307.63	0.1831	αHO→ αLU+1 (0.61046)
				βHO-6→ βLU (0.54166)
				βHO-6→ βLU+1 (-0.36249)
D_{10} (*B*)	4.359	284.38	0.0241	αHO→ αLU+2 (0.85236)

Table S2. Excited states of F_2PyBTM at the D_0 optimized structure. f denotes the oscillator strength.

State	Excitation Energy /eV	/nm	f	Major Configurations (Expansion Coefficients)
D_1 (B)	3.140	394.77	0.0187	βHO-2→ βLU (0.38719)
				βHO-1→ βLU (0.83463)
D_2 (A)	3.229	383.97	0.0281	βHO-3→ βLU (-0.59705)
				βHO→ βLU (0.65565)
D_3 (B)	3.240	382.64	0.0461	βHO-4→ βLU (0.37208)
				βHO-2→ βLU (0.77002)
				βHO-1→ βLU (-0.35058)
D_4 (A)	3.592	345.16	0.0031	βHO-3→ βLU+1 (0.64042)
				βHO→ βLU (0.68143)
D_5 (B)	3.613	343.16	0.0006	βHO-5→ βLU (0.81507)
				βHO-5→ βLU+2 (-0.36597)
D_6 (B)	3.681	336.79	0.0086	βHO-4→ βLU (0.83491)
				βHO-2→ βLU (-0.33610)
D_7 (A)	3.739	331.54	0.0517	αHO→ αLU+1 (0.53545)
				βHO-6→ βLU (0.60619)
D_8 (B)	3.947	314.08	0.2352	αHO→ αLU (0.86142)
D_9 (A)	4.135	299.79	0.3205	αHO→ αLU+1 (0.66057)
				βHO-6→ βLU (-0.49278)
D_{10} (B)	4.355	284.65	0.0172	αHO→ αLU+2 (0.86458)

Table S3. Calculated Faraday B terms of electronic transition from m (D_0) to n for PyBTM. The total value was obtained by summing over k =D_{50}.

	$B_{nm}^{(k)}$ /a.u.			
k	$n = D_1$	$n = D_8$	$n = D_9$	$n = D_{10}$
D_1	--	-0.3905	0.0286	2.8173
D_2	1.5754	0.0000	1.4498	0.9542
D_3	0.6819	8.3228	-0.2065	0.8110
D_4	-0.5149	0.0000	-0.9492	0.1430
D_5	-2.0008	-6.7904	-7.0002	-0.0669
D_6	0.1872	0.0000	-11.7737	-5.9070
D_7	-0.1277	123.8621	3.9961	-12.5786
D_8	0.3905	--	-162.8111	-11.9821
D_9	-0.0286	162.8111	--	-20.7731
D_{10}	-2.8173	11.9821	20.7731	--
D_{11}	-1.0949	0.0000	5.9005	-3.9362
D_{12}	0.1598	0.0000	-0.9122	-0.7573
D_{13}	-0.1766	0.0000	0.6271	0.2984
D_{14}	0.0010	-0.3192	0.0084	-4.9095
D_{15}	-0.0159	0.7640	0.0321	-0.1338
D_{16}	0.0298	-2.0871	-0.2505	-0.8950
D_{17}	0.0121	0.0000	0.0727	0.0975
D_{18}	-0.1902	0.9731	-0.1182	-0.2348
D_{19}	-0.2478	-0.5040	-1.0363	0.0556
D_{20}	-0.2363	0.0000	-1.3251	-0.0699
Total	-2.3208	298.9970	-149.0635	-59.4907

Table S4. Calculated Faraday B terms of electronic transition from m (D_0) to n for F_2PyBTM. The total value was obtained by summing over k =D_{50}.

	$B_{nm}^{(k)}$ /a.u.			
k	$n = D_1$	$n = D_8$	$n = D_9$	$n = D_{10}$
D_1	--	0.0839	0.0702	1.2247
D_2	-3.1551	-2.0963	0.0000	1.1815
D_3	0.8468	-0.4570	6.8511	1.3120
D_4	0.4965	-3.0721	0.0000	0.2972
D_5	-0.0488	0.4668	1.0332	0.1797
D_6	-1.0644	-10.6526	-6.3879	0.0267
D_7	-4.7565	22.1929	0.0000	-2.8639
D_8	-0.0839	--	-81.2100	-28.0276
D_9	-0.0702	81.2100	--	-14.3302
D_{10}	-1.2247	28.0276	14.3302	--
D_{11}	-0.4840	11.9617	0.0000	-4.6887
D_{12}	0.5734	-7.9451	0.0000	0.2070
D_{13}	-0.0364	0.1330	-1.6873	-3.2782
D_{14}	0.4521	0.6385	0.0000	0.4689
D_{15}	0.0139	-0.2051	10.1984	-0.2964
D_{16}	0.0766	-0.0834	0.0000	-0.0222
D_{17}	-0.0478	-0.2447	-1.2509	-0.0858
D_{18}	-0.0602	-1.1803	0.0000	0.0143
D_{19}	0.0029	-0.4560	-4.1325	-0.5190
D_{20}	-0.0454	-0.1819	-2.6578	-0.3063
Total	-7.7447	119.0802	-69.4008	-51.7600
